# Covert Cognition in Disorders of Consciousness: A Meta-Analysis

**DOI:** 10.3390/brainsci10120930

**Published:** 2020-12-02

**Authors:** Caroline Schnakers, Michaela Hirsch, Enrique Noé, Roberto Llorens, Nicolas Lejeune, Vigneswaran Veeramuthu, Sabrina De Marco, Athena Demertzi, Catherine Duclos, Ann-Marie Morrissey, Camille Chatelle, Anna Estraneo

**Affiliations:** 1 Casa Colina Hospital and Centers for Healthcare, Pomona, CA 917697, USA; 2 Department of Psychology, University of California Los Angeles, Los Angeles, CA 90095, USA; hirschm92@gmail.com; 3 NEURORHB, Servicio de Neurorrehabilitación de Hospitales Vithas, 46011 València, Spain; quique@neurorhb.com (E.N.); roberto@neurorhb.com (R.L.); 4 Neurorehabilitation and Brain Research Group, Instituto de Investigación e Innovación en Bioingeniería, Universitat Politècnica de València, 46022 València, Spain; 5 GIGA-Consciousness, Coma Science Group, University of Liège, 4000 Liège, Belgium; nicolas.lejeune@chnwl.be (N.L.); a.demertzi@ulg.ac.be (A.D.); camille.chatelle@uclouvain.be (C.C.); 6 ReGen Rehabilitation Hospital, Petaling Jaya 46200, Malaysia; vicveera@gmail.com; 7 Servicio de Terapia Ocupacional, Clínica Universitaria Reina Fabiola, Universidad Católica de Córdoba, Córdoba X5000 IYG, Argentina; sabri.dmarco@gmail.com; 8 School of Physical and Occupational Therapy, McGill University, Montreal, QC H3A 0G4, Canada; catherine.duclos@mail.mcgill.ca; 9 Ageing Research Centre, Health Research Institute, School of Allied Health, University of Limerick, V94 T9PX Limerick, Ireland; a.morrissey2@gmail.com; 10 IRCCS Fondazione Don Carlo Gnocchi, 50143 Florence, Italy; aestraneo@gmail.com; 11 Neurology Unit, Santa Maria della Pietà General Hospital, 80035 Nola, Italy

**Keywords:** severe brain injury, consciousness, vegetative state, minimally conscious state, covert cognition, cognitive motor dissociation

## Abstract

Covert cognition in patients with disorders of consciousness represents a real diagnostic conundrum for clinicians. In this meta-analysis, our main objective was to identify clinical and demographic variables that are more likely to be associated with responding to an active paradigm. Among 2018 citations found on PubMed, 60 observational studies were found relevant. Based on the QUADAS-2, 49 studies were considered. Data from 25 publications were extracted and included in the meta-analysis. Most of these studies used electrophysiology as well as counting tasks or mental imagery. According to our statistical analysis, patients clinically diagnosed as being in a vegetative state and in a minimally conscious state minus (MCS−) show similar likelihood in responding to active paradigm and responders are most likely suffering from a traumatic brain injury. In the future, multi-centric studies should be performed in order to increase sample size, with similar methodologies and include structural and functional neuroimaging in order to identify cerebral markers related to such a challenging diagnosis.

## 1. Introduction

Disorders of consciousness (DOC) are currently diagnosed based on behavioral profile and defined criteria. Vegetative state (VS) (also named unresponsive wakefulness syndrome) is characterized by the presence of eye opening (and arousal) in the absence of oriented or willful behaviors [1], while minimally conscious state (MCS) is characterized by both eye opening and reproducible, although minimal, oriented and/or willful behaviors (e.g., visual tracking or command following) [2]. MCS has also been subcategorized into two clinical entities, MCS+ and MCS−, based on the presence or absence of language-related behaviors (i.e., command following, intelligible verbalization, and/or intentional communication) [3].

Detecting signs of consciousness and therefore making an accurate diagnosis based on behavioral responses is nevertheless challenging and, as repeatedly shown in the literature, leads to errors in approximately 40% of cases [4]. This high misdiagnosis rate likely reflects several sources of variance [5]. Variance in diagnostic accuracy may result from biases contributed by the patient (e.g., sensory deficits, fatigue, and spasticity), the environment (e.g., positioning, excessive noise, and lack of light) or, even more importantly, the examiner and the way the examiner assesses the patient [6]. Indeed, a series of studies have shown that the type of scale that is used to detect signs of consciousness is essential and that the Coma Recovery Scale—Revised (CRS-R) represents the most valid and sensitive scale currently available for increasing diagnostic accuracy [7].

However, despite the best scales that could be used to decrease the misdiagnosis rate, a different group of patients who are unable to show any behavioral signs of receptive language but are able to respond mentally to active neuroimaging or electrophysiological paradigms has been identified in the last decade. In 2006, Owen and colleagues reported the case of a young woman with severe brain injury classified as being in a vegetative state. When performing a mental imagery task (e.g., imaging playing tennis), her fMRI-related brain activity was similar to the one observed in healthy controls [8]. Following this study, Monti and colleagues have tested 54 patients using the same fMRI paradigm. Two patients clinically diagnosed as being in a VS and three patients clinically diagnosed as being in a MCS were able to perform the task (9% of the sample). One of these patients was able to answer ‘yes’ or ‘no’ to autobiographical questions and therefore communicated by using either motor or spatial imagery [9]. Since then, a series of studies has been published about the detection of willful brain activity in patients who do not show command following at the bedside, confirming the existence of patients with covert cognition (recently named “cognitive motor dissociation” or CMD) [10,11]. One systematic review and meta-analysis on the use of passive and active paradigm in DOC patients found that CMD seems to be less common in VS (14% of cases) than in MCS [12]. However, to date, the overall profile of this new clinical entity has not been systematically investigated yet and is crucially needed to help clinicians in identifying this challenging population.

Therefore, in this metanalysis, the following primary research question was formulated using the Patients, Intervention, Comparison, Outcomes (PICO) approach [13]. In DOC patients (P), does a clinical diagnosis of VS (I) as compared to MCS or MCS− (C) indicate lower odds for a patient to be able to respond to active paradigms (O)? A secondary objective was to identify demographic and other clinical variables such as age, time since injury, etiology and behavioral patterns (as determined by CRS-R total scores and subscores) that might influence whether or not a DOC patient is able to respond to an active paradigm during paraclinical tests (such as functional neuroimaging and/or electrophysiological recordings).

## 2. Materials and Methods

### 2.1. Inclusion and Exclusion Criteria

To be included in this meta-analysis, the following criteria had to be met: (1) observational study (i.e., cross-sectional, longitudinal, retrospective or prospective), (2) human subjects aged 18 years old and above with a VS or a MCS diagnosis, (3) subjects were evaluated using neuroimaging and/or electrophysiological active paradigms (defined as subjects instructed to mentally perform a task), and (4) studies published between 2006 and 2019. All etiologies and all clinical settings were included. Studies that were not published in English were excluded.

For the meta-analysis, studies were included if they had (1) at least 5 human subjects with DOC [12], (2) a diagnosis of VS or MCS established using the CRS-R (excluding data related to a diagnosis of coma or emergence from MCS (characterized by the recovery of functional communication and/or functional object use), (3) data that could be extracted at the single-subject level, and with identifiable responders (and non-responders) to active paradigm.

### 2.2. Search Methods

The meta-analysis was performed in accordance with the Preferred Reporting Items for Systematic Reviews and Meta-Analyses (PRISMA) guideline [14]. Search terms were generated in consultation with the Powell library at the University of California Los Angeles (Supplementary Materials). An electronic search of published studies was performed on PubMed in June 2019. The titles and abstracts of all articles in the search were screened by both MH and CS. Additional articles were manually searched by cross-referencing using the ‘cited by’ function as well as by reviewing the reference section of the selected papers. Relevant articles from this initial screening were then gathered by MH.

Ten raters who are members of the International Brain Injury Association (IBIA) Disorders Of Consciousness Special Interest Group (DOC SIG) were assigned in groups of 2 and independently reviewed the quality of each screened study, using the Quality Assessment of Diagnostic Accuracy Studies-2 (QUADAS-2) [15]. When the review was completed, ratings were shared. In case of divergent responses, a consensus (between both raters) had to be reached and, if needed, was mediated by CS. The QUADAS-2 comprises four domains: (1) participant selection, (2) index test, (3) reference standard, and (4) flow of participants through the study and timing of the index tests and reference standard (flow and timing). Each domain is assessed for risk of bias, and the first three domains are also assessed for concerns regarding applicability (for the target questions used in this study, see Supplementary Materials). Risk of bias and concerns about applicability are judged as ‘low’, ‘high’ or ‘unclear’.

### 2.3. Statistical Analyses

Based on the QUADAS-2, the analysis only included studies with a “low risk of bias” and/or “low concern regarding applicability” [15]. Data of the selected studies were extracted by MH and CS in a excel spreadsheet. When data were not available in the paper, the first and/or last author of the study was contacted. The following variables were collected, when available: clinical diagnosis (VS and MCS), age, time since injury, etiology and CRS-R total scores and subscores. For studies where CRS-R subscores were available, a diagnosis of MCS+ versus MCS− was attributed or confirmed (respectively, presence versus absence of command following, intelligible verbalization, and intentional communication) [3]. The modality of assessment and the type of task used to detect covert cognition were documented.

JASP (free open-source statistic program; https://jasp-stats.org/) was used to perform the meta-analyses. Effect sizes (and their standard error) were estimated for each study and for each of the variables cited above. Restricted Maximum Likelihood was used to account for the weight that each study carries in the compounded meta-analytic estimated effect. Confidence intervals (based on z statistic) were calculated and reported. Forest plots were used to illustrate our results. Finally, publication bias was estimated using Funnel plots and rank correlations for Funnel plot asymmetry (Kendall’s tau).

## 3. Results

**Figure 1:**
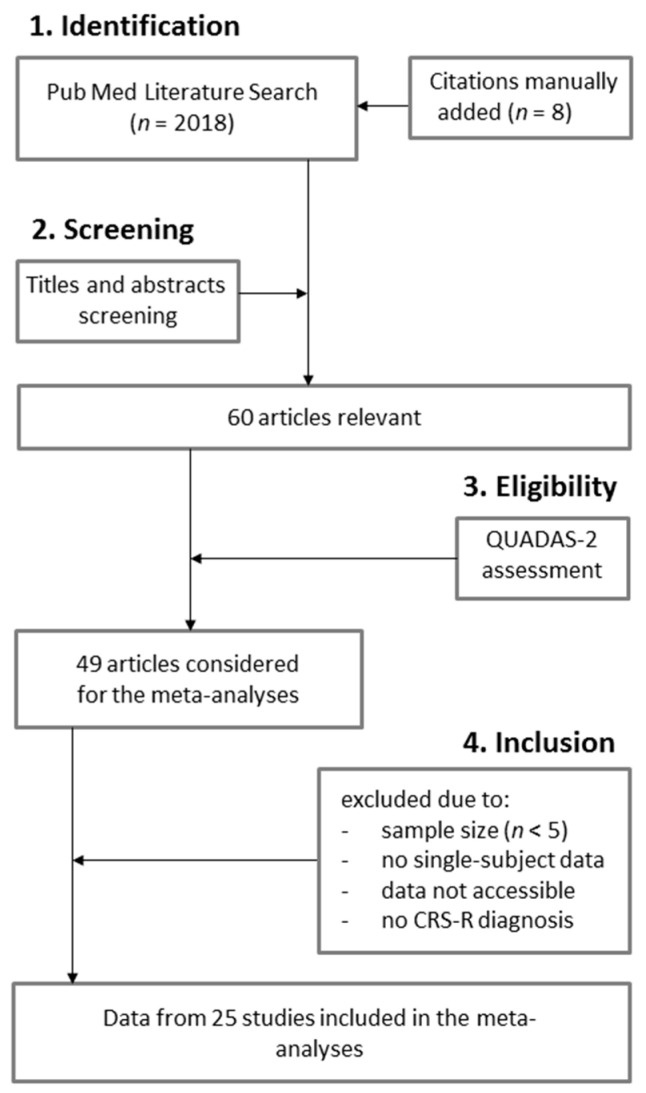
PRISMA Flow Diagram.

Our literature search on PubMed yielded 2018 citations (Figure 1). Eight citations were manually added to our list of articles. Sixty original observational studies were found relevant. Based on the QUADAS-2, 49 studies were considered. However, 24 publications were excluded due to: (1) the sample size (i.e., less than 5 subjects) (*n* = 12), (2) the absence of single-subject data (*n* = 7), (3) the data not being accessible (*n* = 3) or (4) the absence of CRS-R diagnosis (*n* = 2). Data from 25 publications were extracted and included in the meta-analysis (Table 1). Most of these studies used electrophysiology (i.e., EEG/ERP or EMG; *n* = 16), while four studies used neuroimaging (i.e., fMRI) and five studies used both electrophysiology (i.e., EEG/ERP) and neuroimaging (i.e., fMRI). Active paradigms in these studies included either counting tasks (*n* = 12), mental imagery (spatial and/or motor) (*n* = 11), or focused attention (*n* = 2). Most studies had a high risk of bias (72%) but had low applicability concerns (92%).

For the meta-analysis, subject-level data were identified in 592 patients (276 VS—average of 705 days post-injury; and 316 MCS− average of 817 days post-injury), among which there were 156 responders. An average of 32.13% (±18.26%) of responders was estimated across studies with a wide range between 7.69% and 71.43%. Age ranged between 18 and 79 years old (with an average of 41 ± 16). Of the responders included in this analysis (and for who the data were available), 54% (83 out of 153) had a traumatic brain injury, 67% (104 out of 156) were diagnosed with MCS and only 24% (35 out of 145) were in an acute DOC (within 28 days post-injury) [16]. Further, based on the data extracted in the 19 (out of 25) studies which made the distinction between MCS+ and MCS− (11 with CRS-R subscores and 8 with diagnosis only), 134 responders were identified—48 VS, 37 MCS− and 49 MCS+. The results of our statistical analyses are described below. Since the effect size was not possible to calculate for all of the variables of interest in some of the selected studies due to missing data or lack of variance, the number of publication on which each result is based is reported.

**Figure 2:**
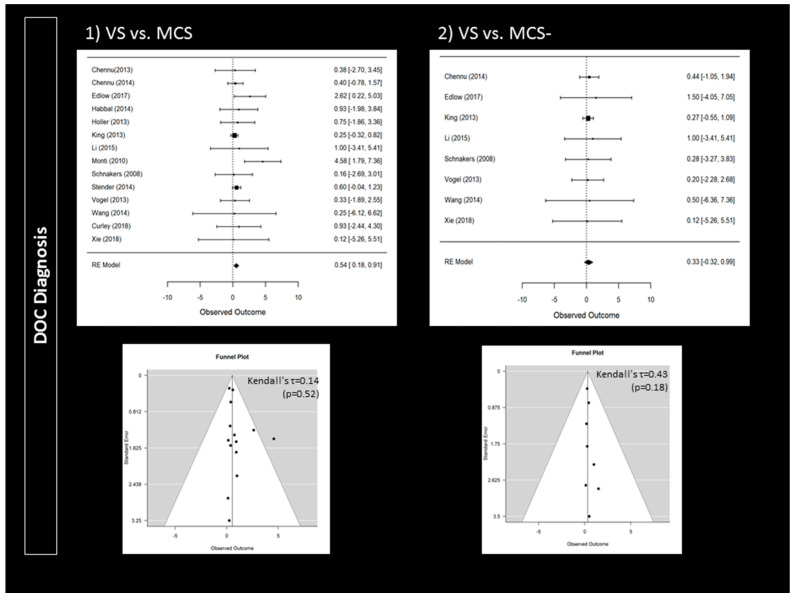
Impact of disorders of consciousness on the rate of responders: forest plots and publication bias (Funnel plots and Kendall’s tau). An observed outcome (and confidence interval) above 0 means MCS > VS (for Panel 1) or MCS− > VS (for Panel 2).

**Figure 3:**
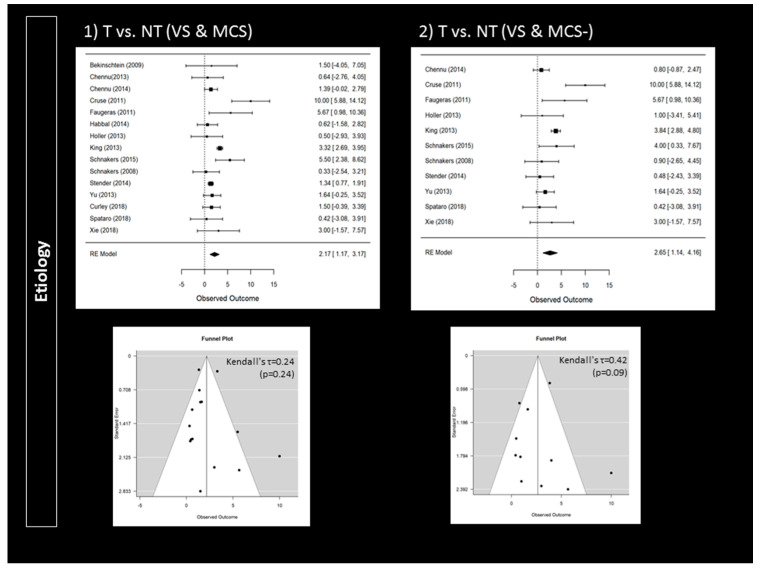
Impact of etiology on the rate of responders: forest plots and publication bias (Funnel plots and Kendall’s tau); Panel 1 illustrates the findings when considering traumatic brain injury (T) versus non-traumatic brain injury (NT) in VS and in MCS patients. Panel 2 illustrates the findings when considering traumatic brain injury (T) versus non-traumatic brain injury (NT) in VS and in MCS− patients. An observed outcome (and confidence interval) above 0 means T > NT (for both Panel 1 and 2).

Compared to patients with a clinical diagnosis of VS, MCS patients were more likely to follow commands during active paradigms (95% CI 0.18 to 0.91; *p* = 0.004; *n* = 14) (Figure 2). Responders were also more likely to present a traumatic brain injury (95% CI 1.17 to 3.17; *p* < 0.001; *n* = 15) (Figure 3). Higher CRS-R total scores (95% CI 0.11 to 0.57; *p* = 0.004; *n* = 23) and higher visual subscores (95% CI 0.06 to 0.56; *p* = 0.014; *n* = 14) were found in responders (Supplementary Materials). Responders did not significantly differ from non-responders regarding age (*n* = 24), time since injury (*n* = 24) or CRS-R subscores (*n* = 14) other than in visual subscores (e.g., auditory subscale). Further analyses only involving patients with a diagnosis of VS or MCS− confirmed that responders more likely suffered a traumatic brain injury (95% CI 1.14 to 4.16; *p* < 0.001; *n* = 11) (Figure 3). However, MCS− patients were not more likely to respond than VS patients (*n* = 8) (Figure 2). Higher CRS-R total scores (*n* = 17) and visual subscores (*n* = 11) were also not found in responders. Based on Funnel plots and Kendall’s tau rank correlations, no significant publication bias was found (Figure 2 and Figure 3 as well as Supplementary Materials).

## 4. Discussion

In this meta-analysis, our main objective was to identify clinical and demographic variables that are more likely to be associated with responding to an active paradigm. Our primary research question was to assess whether responder rate varies according to DOC diagnosis and, further, whether a clinical diagnosis of VS indicates lower odds for a patient to be able to respond to active paradigms as compared to the overall MCS group or only MCS−. According to our results, a diagnosis of MCS seems to be associated with a higher odds than a diagnosis of VS. This is in line with a previous meta-analysis that showed that CMD is less frequent in VS patients [12]. We also report for the first time the likelihood of responding to an active paradigm in MCS−. Indeed, one previous meta-analysis has reported data for MCS as a whole, including patients who show command following at the bedside [12]. We here show that the likelihood of responding to an active paradigm is not higher in MCS− as compared to VS, across studies. This is not surprising, as, by definition, on the contrary to MCS+, patients in a MCS− are unable to show that they understand language (e.g., behavioral response to command). In the literature, the clinical subcategorization of MCS is further supported by metabolic differences in areas that are associated with consciousness (i.e., lower metabolism in precuneus and thalamus in MCS−) [41] and with both receptive and expressive language (i.e., lower metabolism in the left middle temporal cortex and lower connectivity between left angular gyrus and left prefrontal cortex in MCS−) [42,43]. The detection of covert cognition in MCS− suggests that, in a fraction of these patients, receptive language impairment might not be the limiting factor when assessing consciousness at the bedside but expressive language impairments (and hence motor limitations) might be. This stresses the importance of making the distinction between MCS+ and MCS−, and of trying to understand whether/how CMD differs between VS and MCS−, in future studies.

A secondary objective of this meta-analysis was to identify the impact of demographic and other clinical variables (i.e., age, time since injury, etiology and behavioral patterns) on a patient’s ability to respond to an active paradigm. According to our data, etiology matters. Further, patients with traumatic brain injury were more likely to be responders, which is in line with a previous meta-analysis [12]. This suggests that brain lesions related to a traumatic brain injury might be associated with covert cognition. In fact, a recent study described a case report of CMD due to a traumatic brain injury that may be caused by impaired connectivity between the thalamus and the primary motor cortex interfering with the execution of willful motor actions [44]. Even though such findings should be confirmed in more patients, a better understanding of the mechanism of injury related to CMD might help to create a therapy using, for example, neuromodulation, such as transcranial direct current stimulation and/or low intensity focused ultrasound pulsation [45].

Behavioral pattern and, further, the visual subscale of the CRS-R were associated with more likelihood to respond to active paradigms. This is interesting since the auditory subscale would rather have been expected to be associated with a higher response rate. Indeed, this subscale of the CRS-R includes items assessing the presence of receptive language such as response to command. However, this finding might be due to the higher proportion of MCS patients showing oriented visual behavior (both MCS+ and MCS−) rather than response to command (only MCS+). This effect actually disappeared when only taking MCS− in account. We therefore unfortunately do not think that the visual subscale of the CRS-R would be of help when trying to detect CMD.

In fact, most behavioral scales such as the CRS-R largely depend on motor output, which complicates the assessment of CMD patients who might have lesions of key areas of the central nervous system related to motor functions (such as the thalamus) or even the peripheral nervous system (e.g., in case of severe spasticity). Recently, several studies have shown that the Motor Behavioral Tool (MBT) might reveal residual cognition in patients diagnosed as VS by the CRS-R [46,47]. The MBT has been found unaffected by the presence of pitfalls that interfere with sensory and motor afferents (e.g., polyneuropathy, myopathy, myelopathy, and aphasia) or intrinsic brain activity (e.g., epilepsy) [48]. Future studies should therefore further investigate this tool and demonstrate its ability to detect CMD at the bedside, as it might represent a critical clinical tool for clinicians.

There are several limitations to consider in this meta-analysis. This meta-analysis primarily included publications found on PubMed. Future studies could use additional databases (e.g., Ovid, Scopus or Google scholars). Studies by the same authors were included. However, when comparing these studies, patients’ demographics were different, suggesting distinct samples. Data from half of the studies that could be included based on the QUADAS-2 could not be included, most frequently due to small sample size. This warrants an increase in multi-centric studies to increase statistical power and to better understand this peculiar clinical entity. Another limitation is that the studies included in this reviewed involved a minority of patients in the acute stage. Our results might therefore not be representative of this population. Future meta-analyses might try to reach a better balance between acute (<28 days post-injury) and prolonged (>28 days post-injury) DOC [16]. Finally, the available studies are less informative about covert cognition detected using neuroimaging paradigms. Indeed, even though initial studies used neuroimaging, most of the studies included used electrophysiology to detect responders, most likely because electrophysiology is more accessible in a clinical setting, has a lower cost and is easier to implement (bedside assessment). A recent review nevertheless warned about the sensitivity and specificity of electrophysiological paradigms used to detect CMD [27]. Most of the studies included in this meta-analysis used paradigms that were largely validated in the literature such as counting tasks or mental imagery (spatial and/or motor). However, the heterogeneity among studies in terms of methodology, task design, and procedures of analysis calls for a harmonization of protocols in the detection of CMD in order to allow a better comparison among them.

In conclusion, the objective of this meta-analysis is to offer a more exhaustive view of what variables might be of interest when considering a diagnosis of CMD. Our results show that VS and MCS− show similar likelihood to respond to an active paradigm and that responders are most likely suffering a traumatic brain injury. In the future, multi-centric studies should be performed in order to increase sample size, have similar methodologies in assessing covert cognition (e.g., using event-related potentials during counting tasks and a systematic assessment of impairments interfering with sensory-motor output) [27] and include structural and functional neuroimaging in order to identify brain mechanisms and possible targets for therapeutic strategies [45].

## Figures and Tables

**Table 1:** Description of the studies included in our meta-analysis.

ARTICLE	Risks of BIAS	APPLICABILITY Concerns	Paradigm	Modality	Responders
Bekinschtein (2009) [17]	HIGH	LOW	Local-global effect (count)	fMRI/EEG	28.57%
Chennu (2013) [18]	HIGH	LOW	Local-global effect (count)	fMRI/EEG	19.05%
Chennu (2014) [19]	HIGH	LOW	Local-global effect (count)	EEG	43.33%
Cruse (2011) [20]	LOW	LOW	Motor imagery	EEG	20.00%
Cruse (2012) [21]	HIGH	LOW	Motor imagery	EEG	21.05%
Edlow (2017) [22]	LOW	HIGH	Motor imagery	fMRI/EEG	43.75%
Faugeras (2011) [23]	HIGH	LOW	Count target	EEG	9.09%
Gibson (2014) [24]	HIGH	LOW	Spatial and motor imagery	fMRI/EEG	66.67%
Habbal (2014) [25]	LOW	LOW	Motor imagery	EMG	10.53%
Hauger (2015) [26]	HIGH	LOW	Count target	EEG	10.00%
Hauger (2017) [27]	HIGH	LOW	Count target	EEG	62.50%
Holler (2013) [28]	HIGH	LOW	Motor imagery	EEG	35.71%
King (2013) [29]	HIGH	LOW	Count target	EEG	26.14%
Li (2015) [30]	HIGH	LOW	Mental calculation	EEG	33.33%
Monti (2010) [9]	HIGH	LOW	Spatial and motor imagery	fMRI	7.69%
Schnakers (2015) [31]	LOW	LOW	Focus attention	EEG	19.05%
Schnakers (2008) [32]	LOW	LOW	Count target	EEG	27.27%
Stender (2014) [33]	HIGH	LOW	Spatial and motor imagery	fMRI	33.33%
Vogel (2013) [34]	HIGH	LOW	Spatial and motor imagery	fMRI	60.00%
Wang (2015) [35]	HIGH	LOW	Count target	EEG	71.43%
Yu (2013) [36]	HIGH	LOW	Count target	fMRI	11.63%
Chatelle (2018) [37]	LOW	HIGH	Count and motor imagery	EEG	40.00%
Curley (2018) [38]	LOW	LOW	Motor imagery	fMRI/EEG	34.78%
Spataro (2018) [39]	HIGH	LOW	Count target	EEG	30.77%
Xie (2018) [40]	HIGH	LOW	Focus attention	EEG	37.50%

This table reports the risk of bias and applicability concerns based on the QUADAS-2. The paradigm and modality used to detect covert cognition in each study are also described (fMRI = functional magnetic resonance imaging, EEG = electroencephalography, and EMG = electromyogram).

## Supplementary Materials

The following are available online at https://www.mdpi.com/2076-3425/10/12/930/s1, Figure S1: Impact of behavioral patterns: Forest plots and publication bias (Funnel plots and Kendall’s tau), Table S1: QUADAS-2 target questions for risk of bias and applicability concerns.
