# Supplementary material for: Covert Cognition in Disorders of Consciousness: A Meta-Analysis
Source: Brain Sci. 2020 Dec 2;10(12):930. doi: 10.3390/brainsci10120930 (PMC7759773; doi:10.3390/brainsci10120930)
Supplement: Supplementary file 1 [file brainsci-10-00930-s001.zip › brainsci-968970-supplementary.docx]

**Search terms:**

(((((((((((((((((vegetative[Text Word]) OR "unresponsive wakefulness syndrome"[Text Word]) OR persistent vegetative state[MeSH Terms]) OR "minimally conscious"[Text Word]) OR consciousness disorders[MeSH Terms]) OR "disorder of consciousness"[Text Word]) OR "disorders of consciousness"[Text Word]) OR conscious*[Text Word]) OR consciousness[MeSH Terms]) OR aware*[Text Word]) OR coma[MeSH Terms]) OR coma[Text Word]) OR comatose[Text Word]) OR unconsciousness[MeSH Terms]) OR unconscious*[Text Word])) AND (((((((((((((((((electroencephalography[MeSH Terms]) OR electroencephalograph*[Text Word]) OR EEG*[Text Word]) OR "evoked potential"[Text Word]) OR "evoked potentials"[Text Word]) OR "event related potential"[Text Word]) OR "event related potentials"[Text Word]) OR ERP*[Text Word]) OR electrophysiolog*[Text Word]) OR "functional magnetic resonance imaging"[Text Word]) OR magnetic resonance imaging[MeSH Terms]) OR "magnetic resonance imaging"[Text Word]) OR MRI[Text Word]) OR fMRI[Text Word]) OR "functional MRI"[Text Word]) OR neuroimaging[Text Word]) OR "functional neuroimaging"[Text Word])) AND ((((((((((((((((task[Text Word]) OR tasks[Text Word]) OR "active paradigm"[Text Word]) OR count[Text Word]) OR counts[Text Word]) OR counted[Text Word]) OR counting[Text Word]) OR imagery[Text Word]) OR "active condition"[Text Word]) OR "top-down process"[Text Word]) OR "top-down processes"[Text Word]) OR "top-down processing"[Text Word]) OR voluntar*[Text Word]) OR willful*[Text Word]) OR covert*[Text Word]) OR command*[Text Word])

**Figure S1: Impact of behavioral patterns: Forest plots and publication bias (Funnel plots and Kendall's tau)**


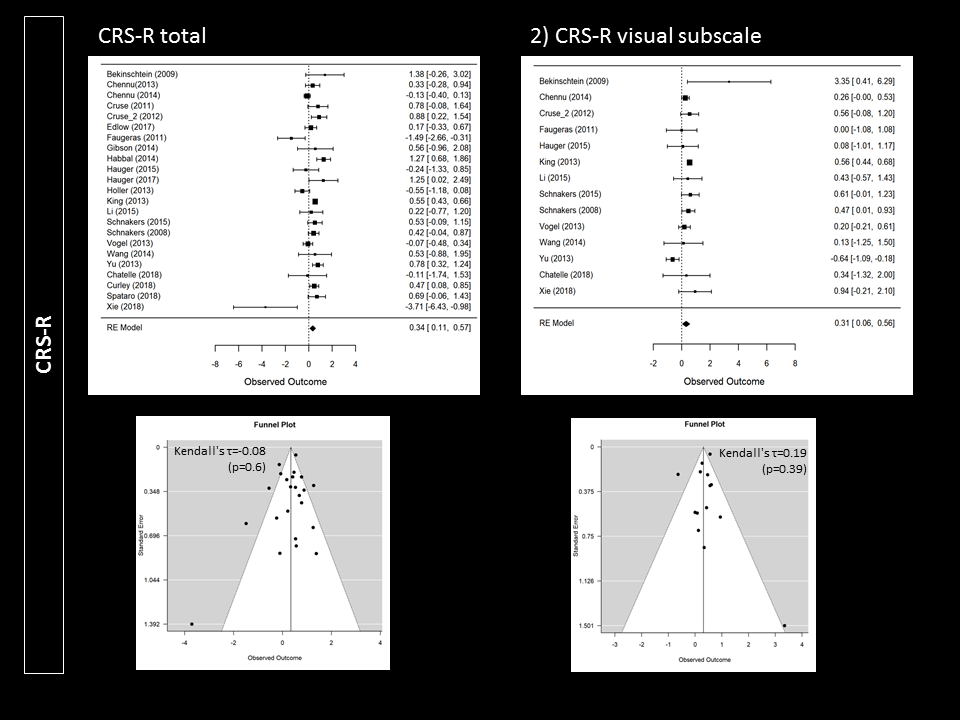


Legend. Panel 1 illustrates the findings when considering the CRS-R total score. Panel 2 illustrates the findings when considering the CRS-R visual subscores.

**Table S1: QUADAS-2 target questions for risk of bias and applicability concerns**

| **Risk of bias** |
| --- |
| ***Patient selection?*** |
| Is there mention of clear exclusion/inclusion criteria? |
| ***Index test:*** |
| Is there a control group? Or, was the paradigm previously validated in healthy controls? |
| ***Reference standard:*** |
| Is the Coma Recovery Scale-Revised used as a diagnostic tool? |
| ***Flow and timing*** |
| Is there an appropriate interval between DOC assessment and the active task? (i.e., within 24h and/or serial assessments) |
| **Applicability concerns** |
| ***Patient selection*** |
| Are they only patients with DOC included in this study? |
| ***Index test*** |
| Is the paradigm appropriate when considering the research question? (ie, active paradigm) |
| ***Reference standard*** |
| Is the diagnostic scale validated to assess DOC? |
